# Childhood meat eating and inflammatory markers: The Guangzhou Biobank Cohort Study

**DOI:** 10.1186/1471-2458-11-345

**Published:** 2011-05-19

**Authors:** CM Schooling, CQ Jiang, TH Lam, WS Zhang, KK Cheng, GM Leung

**Affiliations:** 1 School of Public Health, Li Ka Shing Faculty of Medicine, The University of Hong Kong, Hong Kong SAR, China; 2 Guangzhou Occupational Diseases Prevention and Treatment Centre, Guangzhou Number 12 Hospital, Guangzhou, China; 3 Department of Public Health and Epidemiology, University of Birmingham, UK; 4 School of Public Health, CUNY, New York, USA

**Keywords:** Cardiovascular disease, inflammation, sex, China, developing country, white blood cell count, childhood nutrition

## Abstract

**Background:**

We hypothesized that socio-economic development could, via nutritionally driven levels of pubertal sex-steroids, promote a pro-inflammatory state among men but not women in developing countries. We tested this hypothesis, using recalled childhood meat eating as a proxy for childhood nutrition, in southern China.

**Methods:**

We used multivariable linear regression in the Guangzhou Biobank Cohort Study phase 3 (2006-8) to examine the adjusted associations of recalled childhood meat eating, <1/week (n = 5,023), about once per week (n = 3,592) and almost daily (n = 1,252), with white blood cell count and its differentials among older (≥50 years) men (n = 2,498) and women (n = 7,369).

**Results:**

Adjusted for age, childhood socio-economic position, education and smoking, childhood meat eating had sex-specific associations with white blood cell count and lymphocyte count, but not granulocyte count. Men with childhood meat eating almost daily compared to <1/week had higher white blood cell count (0.33 10^9^/L, 95% confidence interval (CI) 0.10 to 0.56) and higher lymphocyte count (0.16 10^9^/L, 95% CI 0.07 to 0.25). Adjustment for obesity slightly attenuated these associations.

**Conclusion:**

If confirmed, this hypothesis implies that economic development and the associated improvements in nutrition at puberty may be less beneficial among men than women; consistent with the widening sex differentials in life expectancy with economic development.

## Background

In long term industrialized western populations poor childhood conditions are associated with cardiovascular disease [1], although the underlying biological pathway is unclear. Cardiovascular disease may have an inflammatory component [2,3]. Poor childhood conditions are also usually associated with a pro-inflammatory state [4-9]. It is increasingly clear that the social patterning of cardiovascular disease or its risk factors, both traditional and non-traditional, is not universal but epidemiologic stage specific [9-14]. There is often a more marked and longer lasting reversal of the usual social patterning among men than women [9-11] for reasons which have tended to be interpreted mainly in terms of contemporaneous risk factors that differ by sex [11].

A factor that countered the generally protective effect of social advantage among men at the early stages of economic development would explain the observed patterns. Inter-generationally and nutritionally driven increases in the amount, tempo and intensity of pubertal development with macro-economic improvement provide a potential explanation [10,15], because pubertal sex-steroids may have long-term sex-specific effects on fat patterning [16], some lipids [17,18] and immune responsiveness [19-21], which are detrimental among men but sometimes protective among women [16-22]. There is increasing evidence from populations with a recent history of economic development, that growing up in more affluent conditions is positively associated among men, but not women, with central obesity [10], ischemic heart disease [23-26] and inflammation [9]. However, the positive association with inflammation among men may be due to obesity [9]. In a large sample from the developing country setting of southern China, we examined whether a marker of childhood nutrition, positively associated with cognition [27], but sex-specifically associated with central obesity and some lipids [28], also had sex-specific associations with inflammatory markers.

## Methods

### Sources of data

The Guangzhou Biobank Cohort Study is a collaboration between the Guangzhou No. 12 Hospital, the Universities of Hong Kong and Birmingham, and has been described in detail [29]. Recruitment of participants draws from "The Guangzhou Health and Happiness Association for the Respectable Elders", a community social and welfare association unofficially aligned with the municipal government where membership is open to anyone aged 50 years or older for a monthly, nominal fee of 4 Yuan (50 US cents). Recruitment for phase 3 took place from September 2006 to January 2008. About 7% of permanent Guangzhou residents aged 50 years and over are members of "The Guangzhou Health and Happiness Association for the Respectable Elders", of whom 11% enrolled for phase 3 recruitment, and were included if they were capable of consenting, ambulatory, and not receiving treatment modalities which if omitted may result in immediate life threatening risk, such as chemotherapy or radiotherapy for cancer, or dialysis for renal failure. Participants underwent a half-day detailed medical interview, including disease history, and physical examination. The Guangzhou Medical Ethics Committee of the Chinese Medical Association approved the study and all participants gave written, informed consent before participation.

The detailed methods of measurement have been reported [29]. In brief, standing height was measured without shoes to the nearest 0.1 centimeter. Sitting height was measured with the participants sitting on a standard stool; leg length was calculated as the difference between height and sitting height. Weight was measured in light clothing to the nearest 0.1 kilogram. Hip circumference was measured at the greatest circumference round the buttocks below the iliac crest. Waist circumference was measured horizontally around the smallest circumference between the ribs and iliac crest, or at the level of the navel for obese participants. Quantitative haematological analysis was performed using a SYSMEX KX-21 haematology analyzer, from which white blood cell counts, lymphocyte and granulocyte counts were available.

### Exposure assessment

In developing countries long running cohort studies that could provide life long measures are not available, potentially precluding valuable evidence from such settings in the foreseeable future. Instead, in this study childhood nutrition was assessed from a simple question about a notable aspect of childhood nutrition, i.e., meat eating, because this cohort grew up in era and setting where meat consumption was relatively low, highly valued and not constrained by religious beliefs. Recalled childhood meat eating was ascertained by asking participants whether they ate meat as a child with answers in Chinese which translate as "never", "about once a year", "about once a month", "about once a week" or "almost daily". Few (<1%) reported "never" eating meat in childhood, and both "about once a year" and "about once a month" represent occasional meat eating, so we created a 3 point scale: "</1 week", "about once a week" and "almost daily". We cannot validate recalled childhood meat eating against actual childhood intake or maternal recall given the age of our participants. However, we have previously shown that recalled childhood meat eating does have the expected negative association with age of menarche and positive associations with height and childhood socio-economic position [27,28]. Moreover, in other contexts, recalled dietary intake from adolescence has been shown to have some reproducibility and validity [30].

### Outcome measure

White blood cell count was considered, as in other studies, as a marker of a pro-inflammatory state [4], and less well functioning immune system. As we do not have a detailed breakdown of different white blood cell types, such as macrophages, we also considered granulocyte and lymphocyte counts as outcomes because these immune cell sub-populations largely relate to innate and adaptive immunity respectively. They have previously been used as markers of inflammation [31,32]. Sex-steroids may also have differential effects on immune cell sub-populations [20,33].

### Statistical analysis

Multivariable regression was used to assess the adjusted association of recalled childhood meat eating with total white blood cell count and its differentials. We examined whether the outcomes had different associations with recalled childhood meat eating by sex or age from the heterogeneity across strata and model fit assessed from the Akaike Information Criterion for models with and without the relevant interaction term. We also included in the model all other interactions of sex or age, as appropriate, with other confounders that contributed to model fit.

Potential confounders considered were age (5 year age-groups), sex (if appropriate), socio economic position at three life stages (based on parental possession of three relevant items (watch, bicycle and sewing machine) in childhood [10], education and longest held occupation), measures of early life living conditions (leg length and seated height (both continuous)) and lifestyle habits (smoking, alcohol use and physical activity). These were selected for inclusion in the final model on a change in estimate criteria for white blood cell count, on which basis longest held occupation, leg length, seated height, alcohol use and physical activity were dropped. The final model (model 1) included age, childhood socio-economic position (from parental possessions), education and smoking status. Model 2 additionally adjusted for body mass index and waist-hip ratio.

## Results

Of the 10,088 participants, 9,867 (99%) had complete data on inflammatory markers, as well as age, body mass index and waist-hip ratio and were included. There were more women (7,369) than men (2,498), and the women were younger (mean age 59.2 (standard deviation 7.6)) than the men (mean age 63.2 (standard deviation 7.6)). Age ranged from 50 to 96 years, but few participants were older than 75 years.

Table 1 shows that recalled childhood meat eating was positively associated with height and socio-economic position. Table 2 shows that the associations of childhood meat eating with total white blood cell count or its differentials did not differ with age, but did differ by sex for total white blood cell count and lymphocyte count.

**Table 1:** Participant Characteristics by Recalled Childhood Meat Eating in 9,867 Older Chinese Men (2,498) and Women (7,369) in Phase 3 Of The Guangzhou Biobank Cohort Study (2006-8).

		Men			Women		
		Recalled childhood meat eating			Recalled childhood meat eating		
		<1/week (n = 1337)	About once a week (n = 878)	Almost daily (n = 283)	<1/week (n = 3686)	About once a week (n = 2714)	Almost daily (n = 969)
Age (yrs): mean (standard deviation (SD))		64.3 (7.6)	61.6 (7.3)	62.5 (7.9)	60.7 (7.9)	57.5 (6.8)	57.9 (7.4)
Height (cm): mean (SD)		163.9 (6.0)	164.8 (5.9)	165.2 (6.3)	153.4 (5.5)	154.5 (5.4)	154.5 (5.4)
Body mass index: mean (SD)		23.4 (3.2)	23.5 (3.0)	23.9 (3.3)	24.1 (3.5)	23.7 (3.3)	24.0 (3.2)
Waist-hip ratio: mean (SD)		0.90 (0.07)	0.90 (0.06)	0.91 (0.07)	0.86 (0.07)	0.84 (0.07)	0.85 (0.06)
Childhood socio-economic position (parental possessions) ^#^	Low	81.2	53.0	35.0	73.6	41.2	23.3
	Medium	10.0	21.6	16.6	13.9	23.6	20.6
	High	7.1	23.6	46.6	9.9	32.3	53.2
	Unknown	1.7	1.8	1.8	2.6	3.0	2.9
Highest educational attainment	< primary	4.0	0.5	0.7	17.9	4.1	3.5
	Primary	32.3	20.3	17.3	38.6	22.8	18.6
	Junior middle	32.0	33.0	24.7	23.0	30.3	29.0
	Senior middle	20.4	29.1	33.2	17.0	34.6	38.5
	Junior college	7.0	11.2	12.0	2.7	6.8	8.0
	College	4.4	5.9	12.0	0.8	1.4	2.6
Smoking status	Never	34.0	41.5	38.5	96.1	97.4	96.7
	Ex-smoker	29.1	22.3	26.5	1.8	0.6	1.0
	Current	35.9	33.9	34.3	1.5	1.4	0.9
	Unknown	1.0	0.3	0.7	0.5	0.6	1.3

# These correspond to parental possession of none (low), 1 to 2 (medium) and 3 (high) of: sewing machine, watch and bicycle

**Table 2:** Akaike Information Criterion values‡ for Models with and without Interactions by Sex and Age in 9,867 Older Chinese Men (2,498) and Women (7,369) in Phase 3 of the Guangzhou Biobank Cohort Study (2006-8).

		All		Men		Women	
	Model †	Interaction by sex		Interaction by age		Interaction by age	
		with	without	with	without	with	without
White blood cell count	1	36632	36634	9662	9656	26958	26946
	2	35981	35980	9565	9558	26417	26404
							
Lymphocyte count	1	17899	17901	4779	4774	13130	13111
	2	17360	17365	4700	4692	12681	12663
							
Granulocyte count	1	31907	31905	8610	8603	23251	23241
	2	31545	31542	8568	8561	22947	22936

†Model 1 adjusted for age, smoking, parental possessions and education

Model 2 additionally adjusted for body mass index and waist-hip ratio

‡A lower Akaike Information Criterion value indicates a better fitting model

Table 3 shows that among men, childhood meat eating was positively associated with total white blood cell count and lymphocyte count in model 1. The associations were attenuated slightly by additional adjustment for body mass index and waist-hip ratio in model 2. In contrast, among women childhood meat eating was not clearly associated with total white blood cell count, lymphocyte count or granulocyte count.

**Table 3:** Adjusted Associations of Recalled Childhood Meat Eating with Inflammatory Markers in 9,867 Older Chinese Men (2,498) and Women (7,369) in Phase 3 of the Guangzhou Biobank Cohort Study (2006-8).

		Mean and (standard deviation)		Recalled childhood meat eating				
	sex		Model †	<1/week	About once a week		Almost daily	
					β	95% CI	β	95% CI
White blood cell count (10^9^/L)	men	6.8 (1.72)	1	reference	0.02	-0.13 to 0.17	0.33	0.10 to 0.56
			2	reference	0.02	-0.13 to 0.16	0.27	0.04 to 0.49
								
	women	6.3 (1.52)	1	reference	-0.03	-0.11 to 0.05	0.09	-0.02 to 0.21
			2	reference	0.01	-0.07 to 0.09	0.09	-0.02 to 0.20
								
Lymphocyte count (10^9^/L)	men	2.17 (0.65)	1	reference	0.05	-0.01 to 0.10	0.16	0.07 to 0.25
			2	reference	0.04	-0.01 to 0.10	0.15	0.06 to 0.23
								
	women	2.18 (0.59)	1	reference	0.003	-0.03 to 0.03	0.04	-0.004 to 0.09
			2	reference	0.02	-0.01 to 0.05	0.04	-0.004 to 0.08
								
Granulocyte count (10^9^/L)	men	4.18 (1.38)	1	reference	-0.01	-0.13 to 0.11	0.18	-0.01 to 0.36
			2	reference	-0.01	-0.13 to 0.11	0.14	-0.04 to 0.32
								
	women	3.72 (1.18)	1	reference	-0.02	-0.09 to 0.04	0.06	-0.04 to 0.15
			2	reference	0.002	-0.06 to 0.06	0.05	-0.04 to 0.14

†Model 1 adjusted for age, smoking, parental possessions and education

Model 2 additionally adjusted for body mass index and waist-hip ratio

## Discussion

In a large study from an understudied non-western developing population, we found that a marker of early life conditions, i.e., recalled childhood meat eating, had sex-specific associations with some inflammatory markers. More frequent childhood meat eating, adjusted for childhood socio-economic position, was positively associated with white blood cell count and lymphocyte count among men but not women.

Despite using a large study, there are caveats. First, we used self-report of childhood meat eating as a proxy for childhood nutrition, which is undoubtedly subject to measurement error. Random misclassification most likely makes the results conservative, for which our large sample size compensates. Recall bias is also possible, although the participants were unlikely to have been aware of their inflammatory status, nor is it obvious why such status should affect their recall of childhood events. As would be expected, recalled childhood meat eating was strongly positively associated with childhood socio-economic position, education and height. Second, our participants were not a randomly selected, population representative sample; however that should not affect internal associations, unless we missed people with specific combinations of childhood meat eating and inflammatory markers. Third, survivor bias is possible, in which case we would have expected differences in associations by age, of which there was no evidence. Fourth, a single measurement of total white blood cell count and its differentials might not reflect long-term immune function. However, white blood cell count is used as a marker of immune status in clinical settings. Within the normal range, white blood cell count is associated with cardiovascular mortality [3]. Fifth, white blood cell count or its differentials could be affected by acute infection or trauma, however people in such a condition would have been unlikely to participate in our study. Finally, we did not adjust for potential confounding by diet, because of the difficulty of obtaining reliable and accurate dietary data in large-scale studies of free-living participants, particularly amongst the Chinese who share several dishes during a meal making individual intake difficult to gauge.

Childhood conditions, in long-term developed countries, are usually consistently negatively associated with markers of a pro-inflammatory state [4-8]. However, a study from a similarly middle income country [9] also found that childhood living conditions had sex-specific associations with an inflammatory marker that were less favourable among men. In the United States, early life socio-economic position has been observed to have a negative association with infectious pathogens among women but not men [34]. Our findings, of childhood meat eating positively associated with some potential markers of a pro-inflammatory state among men but mot women, has some commonality with these later two studies [9,34]. Our study went further by specifically examining a measure of childhood nutrition, recalled childhood meat eating, adjusted for childhood socio-economic position.

There are several possible explanations for our observations. Childhood meat eating could be a marker of a generally unhealthy childhood diet, including fast food, trans fats and a low fruit and vegetable intake, with corresponding consequences. However, our participants spent their lives in China. Their childhood in China spanned the mid-20^th ^century when living standards were low, and diet was very limited with no fast food and little trans fat [35,36]. More frequent meat eating in our population almost certainly represents a less limited diet, which is consistent with the taller height and earlier age of menarche amongst those with more frequent recalled childhood meat eating [27,28].

Alternatively, we could place these observations within a theoretical hypothesis driven framework [15,37]. Specifically, pubertal sex-steroids are nutritionally driven [38-40], either by meat eating or some other aspect of a less limited diet, with potentially long-term effects. Testosterone suppresses immune responsiveness, while estrogen may promote it [19-21]. Higher sex-steroids might be expected to result in a more pro-inflammatory state among men but not women. Whether a greater impact would be expected on lymphocytes than granulocytes remains to be determined, as do any other possible consequences for immunity or auto-immunity. As such, this hypothesis potentially provides an explanation for several previous observations [9,34] as well as ours; nevertheless it remains speculative because pubertal testosterone is not available in this study. Moreover, our study cannot establish causality; merely provide evidence consistent with a hypothesis.

## Conclusions

Protective effects of better childhood conditions may be less evident among men than women at the early stages of economic development, perhaps due to the biological consequences of up-regulation of the gonadotropic axis. Fully delineating the intergenerational and life long biological effects of changes in nutrition with economic development could be key to developing effective interventions and ensuring that men benefit as much as women from economic development.

## Competing interests

The authors declare that they have no competing interests.

## Authors' contributions

THL, CQJ and KKC initiated and oversee the Guangzhou Biobank Cohort Study, WSZ and GML assisted in the planning and co-ordination of the study. CMS designed this analysis. GML contributed to the interpretation of this analysis. CMS drafted the manuscript. All authors critically reviewed the manuscript for intellectual content. All authors read and approved the final manuscript.

## Pre-publication history

The pre-publication history for this paper can be accessed here:

http://www.biomedcentral.com/1471-2458/11/345/prepub
